# Supplementary material for: Conditional chemoconnectomics (cCCTomics) as a strategy for efficient and conditional targeting of chemical transmission
Source: eLife. 2024 Apr 30;12:RP91927. doi: 10.7554/eLife.91927 (PMC11060718; doi:10.7554/eLife.91927)
Supplement: Figure 3—source data 3. [file elife-91927-fig3-data3.zip › Figure 3 source data3 part 1.zip!/Figure 3-Source data 3-part1/1081-1-CRISPResso_Report_8zosk6yj.zip!/CRISPResso_on_1081_4/2b.Nucleotide_percentage_quilt_around_sgRNA_tttctggactacagtcccca.pdf]

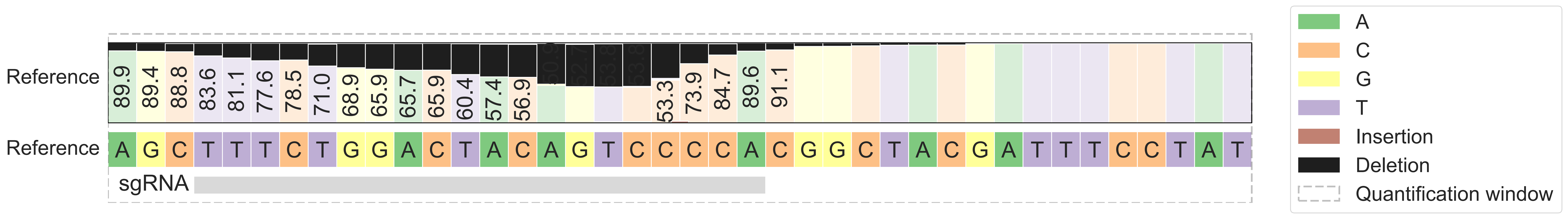
Reference
89.9
89.4
88.8
83.6
81.1
77.6
78.5
71.0
68.9
65.9
65.7
65.9
60.4
57.4
56.9
53.3
73.9
84.7
89.6
91.1
Reference
A G C T T T C T G G A C T A C A G T C C C C A C G G C T A C G A T T T C C T A T
sgRNA
A
C
G
T
Insertion
Deletion
Quantification window
